# Effect of Position Change After Induction of Spinal Anesthesia with Hyperbaric 0.5% Bupivacaine on Duration of Analgesia and Opioid Demand in Percutaneous Nephrolithotomy Candidates

**DOI:** 10.5812/aapm-153617

**Published:** 2025-01-21

**Authors:** Alireza Jaffari, Homayoun Aghamohammadi, Masoud Forouzmehr

**Affiliations:** 1 Anesthesiology Research Center, Imam Hossein Hospital, Shahid Beheshti University of Medical Sciences, Tehran, Iran; 2 Department of Anesthesiology, School of Medicine, Shahid Beheshti University of Medical Sciences, Tehran, Iran; 3 Anesthesiology Research Center, Labbafinejad Hospital, Shahid Beheshti University of Medical Sciences, Tehran, Iran

**Keywords:** Spinal Anesthesia, Analgesia, Opioid, Percutaneous Nephrolithotomy, Position

## Abstract

**Background:**

Post-induction positioning influences the onset speed of the sensory block by affecting anesthetic distribution. Techniques such as using opioids and extending recovery stays aim to enhance this process.

**Objectives:**

This study aimed to evaluate the impact of transitioning patients from a sitting to a lateral position immediately after the induction of 0.5% hyperbaric bupivacaine spinal anesthesia on postoperative pain and opioid consumption.

**Methods:**

In this prospective, randomized clinical trial, patients scheduled for percutaneous nephrolithotomy (PCNL) under spinal anesthesia at Shahid Labafinejad Hospital in 2023 were divided into intervention (lateral position) and control (supine position) groups. Blood pressure, mean arterial pressure (MAP), and heart rate were recorded upon entering recovery, then every 10 minutes up to 60 minutes, and every 15 minutes up to 120 minutes post-operation. Pain levels were assessed using the Visual Analogue Scale (VAS) at specified intervals. Patient satisfaction with analgesia quality was also evaluated.

**Results:**

The study included 35 patients in the lateral group and 34 in the supine group. Pain levels significantly differed between the groups over time (P = 0.0001). The lateral group had a longer analgesia duration (28.8 ± 10.0 minutes vs. 22.9 ± 2.9 minutes, P = 0.105) and lower total narcotic consumption (21.7 ± 5.8 mg vs. 30.4 ± 10.2 mg, P = 0.012). Mean arterial pressure changes showed no significant difference (P = 0.061). Patient satisfaction was significantly higher in the lateral group (P = 0.0001).

**Conclusions:**

Transitioning from the sitting to lateral position post-induction with hyperbaric bupivacaine enhances hemodynamic stability, improves drug distribution in the cerebrospinal fluid (CSF), and enhances sensory block quality. This approach increases postoperative analgesia duration, reduces opioid use and related complications, and decreases the duration of surgery.

## 1. Background

Postoperative pain management remains a significant challenge despite advancements in anesthesia techniques. Opioids, commonly used for pain relief, carry adverse effects and potential risks (1-3). Spinal anesthesia is frequently preferred for procedures such as urological, abdominal, and lower limb surgeries due to its safety profile, rapid onset, and effectiveness in pain management (4, 5). Enhancing postoperative pain control, minimizing recovery time, reducing nausea and vomiting, and optimizing analgesic requirements are critical goals (6). 

Patient positioning immediately after spinal anesthesia induction influences the distribution of anesthetics in the cerebrospinal fluid (CSF), which affects the onset of sensory and motor blocks. Studies have shown that altering patient position following spinal injection with hyperbaric bupivacaine can improve the efficacy of sensory blocks, although it may pose risks such as neurovascular compression (5, 7). The impact of patient positioning on hemodynamic stability and sensory block onset has yielded mixed findings in previous research (8). 

Optimal anesthesia techniques aim to minimize pain, recovery time, nausea, and additional analgesia needs. Spinal anesthesia complications depend on factors such as needle type, drug dose, patient characteristics, and positioning. Post-spinal positioning affects drug distribution and block effectiveness, with lateral positioning potentially improving sensory blocks but risking neurovascular compression (8-11). 

Research on the impact of patient positioning on hemodynamic stability and sensory block onset shows conflicting results (12-14). Despite promising findings, there is limited understanding of how regional anesthesia and patient positioning affect opioid use. Specific multimodal approaches to pain management have shown potential to enhance postoperative pain control (12, 15). 

Given the critical role of opioids in surgical care, further investigation into the effects of spinal anesthesia and patient positioning on postoperative pain and complications is crucial. Few studies have specifically examined these impacts, highlighting the need for more research in this area.

## 2. Objectives 

The study aimed to optimize drug distribution in the CSF, enhance sensory block efficacy, prolong analgesic duration, minimize opioid use and side effects, ensure hemodynamic stability, and improve patient satisfaction. Specifically, it examined the effects of transitioning patients from a sitting to a lateral position immediately after spinal anesthesia induction with hyperbaric bupivacaine on postoperative pain management and opioid consumption.

## 3. Methods

### 3.1. Study Design 

This prospective, randomized clinical trial was conducted in 2023 at Labafinejad Hospital, with Clinical Trial Registration number IRCT20221109056450N1. Eligible participants were all adults scheduled for percutaneous nephrolithotomy (PCNL) under spinal anesthesia during the study period.

### 3.2. Inclusion/Exclusion Criteria 

Inclusion criteria were adults aged 18 years and older, who consented to participate and were candidates for PCNL under spinal anesthesia. Exclusion criteria included: Unwillingness to participate; neurological diseases; diabetes; hypertension; cardiovascular diseases; history of substance abuse; lower limb vascular disorders; prior spinal surgery; neurological, neuromuscular, or psychiatric diseases; chronic pain syndromes; contraindications to spinal anesthesia; recent opioid or NSAID use; narcotic injection during spinal anesthesia; and pregnancy.

### 3.3. Procedure 

After noninvasive monitoring, patients were randomly assigned to either the intervention group (lateral position) or the control group (supine position). Spinal anesthesia was administered in the sitting position at the L3-L4 or L4-L5 level using a 25-gauge Quincke needle with 15 - 20 mg of 0.5% hyperbaric bupivacaine. The intervention group (lateral position) and the control group (supine) were not blinded. Onset time was assessed using dermatome testing, and surgeries were initiated after confirming sensory block and ensuring hemodynamic stability with adequate roll-padding.

### 3.4. Outcome Measures 

Hemodynamic instability was defined as a > 30% drop in systolic blood pressure from baseline or a systolic pressure < 100 mmHg, which was managed with intravenous fluids and ephedrine. A heart rate < 60 bpm prompted the administration of intravenous atropine. Postoperative monitoring involved measuring blood pressure, mean arterial pressure (MAP), and heart rate at specified intervals. Pain levels were assessed using the Visual Analogue Scale (VAS), with VAS > 3 prompting intramuscular pethidine administration. The duration of analgesia was defined as the time from onset to the first pain complaint. Patient satisfaction was recorded at discharge using VAS. Any complications were documented as they occurred.

### 3.5. Statistical Analysis 

Data were analyzed using SPSS version 19. The normality of the data was assessed using the Smirnov-Kolmogorov test. Quantitative variables were compared using the *t*-test, Mann-Whitney test, repeated measures ANOVA, and paired *t*-test. Qualitative variables were evaluated using the chi-square test. Statistical significance was set at P < 0.05.

## 4. Results 

### 4.1. Demographic Comparison 

There were no significant differences in age, weight, or sex between the groups (Table 1). 

**Table 1.** Comparison of Demographic Information Between the Two Groups ^a^

Variables	Lateral Group (n = 35)	Supine Group (n = 34)	P-Value
**Age (y)**	49.8 ± 11.2	49.4 ± 9.0	0.871
**Weight (kg)**	80.4 ± 7.0	80.2 ± 7.3	0.870
**Gender**			0.364
Male	28 (80.0)	24 (70.6 )	
Female	7 (20.0 )	10 (29.4 )	

^a^ Values are expressed as mean ± SD or No. (%).

### 4.2. Hemodynamic Stability 

Significant differences were observed in systolic blood pressure changes (P = 0.015) and heart rate changes (P = 0.006) between the groups. However, no significant differences were found in diastolic blood pressure (P = 0.522) or MAP (P = 0.061) changes.

**Figure 1.**
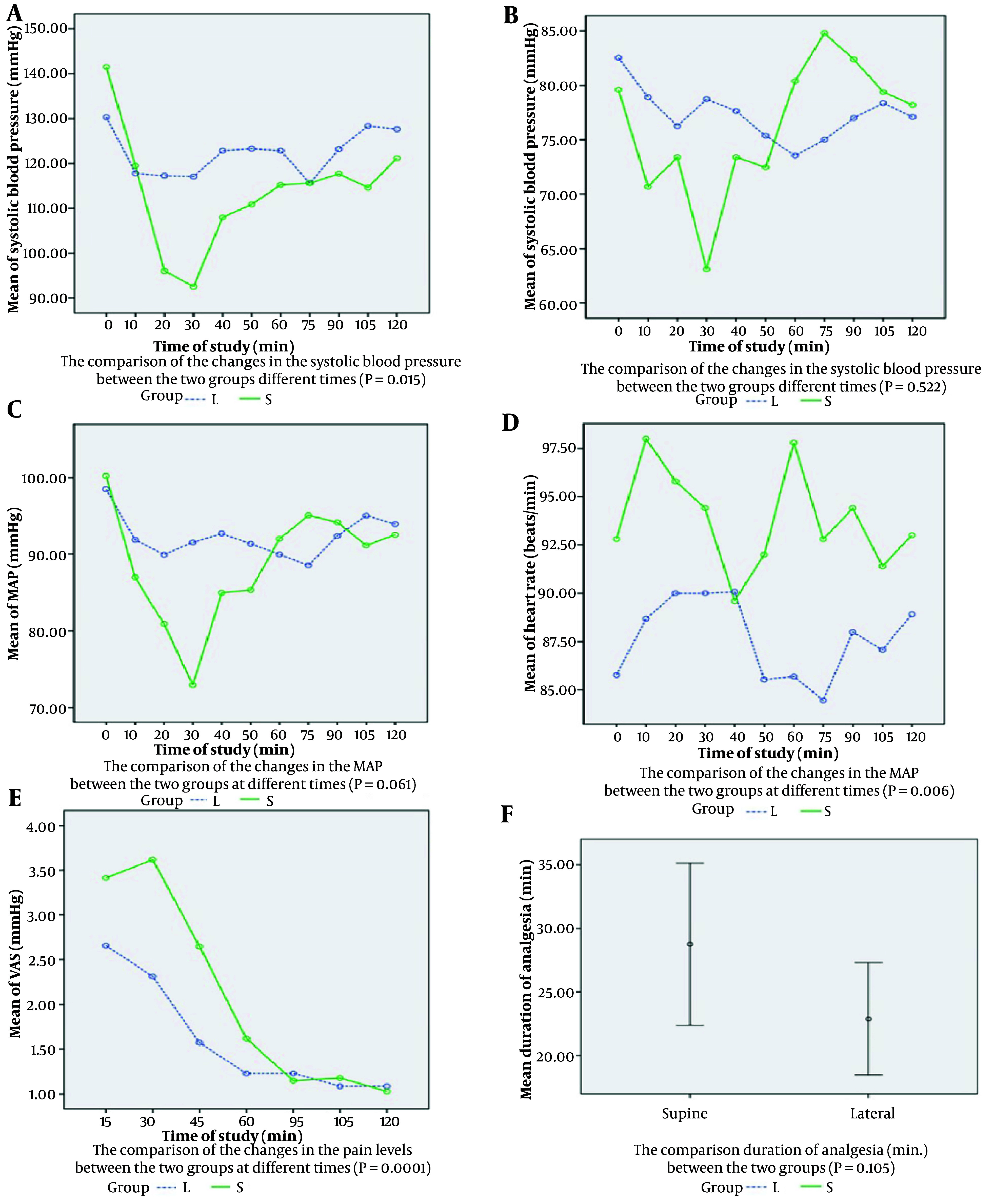
A - F, Statistical analysis between the two groups at different times

Comparisons between the two groups regarding systolic blood pressure changes at 10-minute intervals up to 60 minutes and at 15-minute intervals up to 120 minutes post-operation are shown in Figure 1A, which demonstrates statistically significant differences (P = 0.015). Diastolic blood pressure changes during the same recovery periods are depicted in Figure 1B, indicating no significant difference between the groups (P = 0.522). Similarly, changes in MAP during these intervals are shown in Figure 1C, with no significant difference observed (P = 0.061). Changes in heart rate during recovery times are presented in Figure 1D, revealing statistically significant differences between the groups (P = 0.006). Changes in pain levels assessed via the VAS at 15, 30, 45, 60, 95, 105, and 120 minutes post-operation are illustrated in Figure 1E, demonstrating statistically significant differences between the groups (P = 0.0001). Furthermore, the mean duration of analgesia in the lateral group (28.8 ± 10.0 minutes) compared to the supine group (22.9 ± 2.9 minutes) showed no significant difference (P = 0.105), as shown in Figure 1F. 

### 4.3. Pain and Analgesia 

Significant differences in pain levels over time were observed (P = 0.0001). The lateral group experienced a longer duration of analgesia (28.8 ± 10.0 minutes vs. 22.9 ± 2.9 minutes, P = 0.105) and lower total opioid consumption (21.7 ± 5.8 mg vs. 30.4 ± 10.2 mg, P = 0.012). Pain levels and opioid consumption at specified intervals showed significant differences, particularly within the first 30 minutes post-operation.

The comparison of data during and after the operation between the two groups is shown in Table 2. The total opioid dose differed significantly between the groups (P = 0.012).

**Table 2.** Comparison of Data During and After the Operation Between the Two Groups ^a^

Variables	Lateral Group (n = 35)	Supine Group (n = 34)	P-Value
**Duration of surgery (min)**	74.9 ± 15.0	77.8 ± 12.9	0.279
**Duration of analgesia (min)**	28.8 ± 10.0	22.9 ± 9.2	0.105
**Total dose of opioid consumption (mg)**	21.7 ± 5.8	30.4 ± 10.2	0.012

^a^ Values are expressed as mean ± SD.

The comparison of the total opioid consumption (mg) at 15, 30, 45, 60, 95, 105, and 120 minutes post-operation between the two groups is shown in Table 3, indicating statistically significant differences at 15 and 30 minutes post-operation (P < 0.05). Additionally, pain levels at 15, 30, 45, and 60 minutes post-operation differed significantly between the two groups, as shown in Table 3 (P < 0.05).

**Table 3.** Comparison of Total Opioid Consumption (mg) vs. Pain Levels Between the Two Groups at Different Times ^a^

Post-operative Time (min)	Opioid Consumption (mg)			Pain Levels		
	Lateral Group (n = 35)	Supine Group (n = 34)	P-Value	Lateral Group (n = 35)	Supine Group (n = 34)	P-Value
**15**	2.3 ± 6.5	7.6 ± 9.9	0.010	2.7 ± 1.3	3.4 ± 1.6	0.021
**30**	4.0 ± 8.1	8.8 ± 10.9	0.033	2.3 ± 1.8	3.6 ± 1.3	0.0001
**45**	1.1 ± 4.7	3.5 ± 7.7	0.124	1.6 ± 0.8	2.6 ± 1.1	0.0001
**60**	0.0 ± 0.0	0.6 ± 3.4	0.310	1.2 ± 0.4	1.6 ± 0.9	0.038
**95**	0.0 ± 0.0	0.0 ± 0.0	1.0	1.2 ± 0.5	1.1 ± 0.4	0.594
**105**	0.0 ± 0.0	0.0 ± 0.0	1.0	1.0 ± 0.3	1.2 ± 0.5	0.294
**120**	0.0 ± 0.0	0.0 ± 0.0	1.0	1.0 ± 0.3	1.0 ± 0.3	-

^a^ Values are expressed as mean ± SD.

In the lateral group, pain intensity remained mild throughout the postoperative period, whereas in the supine group, pain was moderate within the first 45 minutes and mild from 60 to 120 minutes post-operation.

Complications, specifically postoperative nausea and vomiting, occurred in 6 (17.1%) patients in the lateral group and 9 (26.5%) in the supine group, with no significant difference between the groups (P = 0.348). However, the incidence of vomiting was significantly lower in the lateral group 45 minutes after surgery, showing a statistically significant difference (P = 0.042). Both groups reported one case of nausea 30 minutes after surgery, with no significant difference between them (P = 0.983).

### 4.4. Patient Satisfaction 

Patient satisfaction was significantly higher in the lateral group (P = 0.0001). Table 4 presents a statistically significant difference (P = 0.0001) in patient satisfaction levels between the two groups.

**Table 4.** Comparison of Patient Satisfaction Levels Between the Two Groups ^a^

Variables	Lateral Group (n = 35)	Supine Group (n = 34)	P-Value
**Unsatisfied **	0 (0 )	0 (0 )	0.0001
**Moderate**	5 (14.3 )	1 (2.9 )	
**Good**	20 (57.1 )	23 (97.1 )	
**Excellent**	10 (28.6 )	0 (0)	

^a^ Values are expressed as No. (%).

### 4.5. Complications 

No significant difference in nausea and vomiting between the groups (P = 0.348), although vomiting was significantly lower in the lateral group 45 minutes post-operation (P = 0.042).

## 5. Discussion

### 5.1. General Analysis 

This study is the first to explore the effects of transitioning patients from a sitting to a lateral position immediately after spinal anesthesia with 15 - 20 mg of 0.5% hyperbaric bupivacaine to improve postoperative pain management and reduce opioid use. The lateral group showed a significant decrease in pain within 60 minutes post-operation, with pain remaining mild. In contrast, the supine group's pain peaked moderately at 45 minutes but decreased to mild from 60 to 120 minutes. No patients reported pain at 120 minutes. Hyperbaric bupivacaine settles more slowly in the lateral position, leading to faster anesthesia onset and a longer duration of analgesia.

### 5.2. Post-induction Positioning Effects 

Patient positioning immediately after spinal anesthesia significantly impacts clinical outcomes. Transitioning from the sitting to the lateral position consistently improves hemodynamic stability, enhances sensory block quality, and reduces hypotension, as supported by our findings and previous research (16, 17). 

Factors such as age, height, anesthetic concentration, needle direction, repeated drug induction, and positioning all affect the extent of sensory and motor block (18). 

Russell et al. demonstrated that lateral positioning leads to more predictable block heights and better surgical conditions compared to other positions, such as the sitting or Oxford positions (16). This supports our observation of reduced intraoperative discomfort and enhanced surgical conditions during cesarean sections. Studies by Kelly et al. and Patel et al. have also emphasized how posture affects the spread and effectiveness of hyperbaric bupivacaine, highlighting the importance of optimal positioning for achieving desired anesthetic effects (5, 7).

### 5.3. Comparison with Prior Studies 

Our study aligns with existing literature suggesting that immediate lateral positioning post-induction significantly reduces pain levels and opioid consumption in the early postoperative period (12, 14). This underscores the benefits of using hyperbaric 0.5% bupivacaine for spinal anesthesia induction and emphasizes the importance of optimal patient positioning to improve recovery outcomes and prolong analgesic effects (19). 

One study demonstrated that, following the induction of spinal anesthesia for arthroscopic knee surgery, both the onset time of motor block and sensory block were shorter in the lateral position compared to the sitting position (20). 

Another study indicated that, for cesarean surgery, the sitting or left lateral positions during spinal anesthesia do not affect the onset time of anesthesia (21). 

Additionally, a study showed that the onset of spinal anesthesia was faster and significantly more pronounced in patients who were placed in the supine position immediately after the subarachnoid block, compared to those who remained in the sitting position for 30 seconds (22). 

The use of hyperbaric bupivacaine in various positions such as lateral, sitting, and Oxford has been extensively studied. These studies have investigated the impact of different positions on the spread and efficacy of spinal anesthesia (8, 16). The results consistently show that lateral positioning leads to a more reliable and higher sensory block level, which correlates with improved intraoperative conditions and a reduced need for supplementary analgesics.

### 5.4. Practical Implications 

Implementing a standardized approach to patient positioning post-induction could potentially optimize the effectiveness of spinal anesthesia. This approach not only improves the quality of anesthesia but also enhances patient comfort and satisfaction (13, 14). By minimizing the risk of inadequate block height or uneven distribution of local anesthetics in the CSF, clinicians can achieve more predictable outcomes in surgical settings. 

The advantage of using a hyperbaric local anesthetic solution lies in the ability to control the level of sensory block by adjusting the patient's position. However, the optimal duration of sitting required to limit the level of sensory block remains controversial. To date, various studies have reported differing results regarding the extension of sensory block in spinal anesthesia.

For instance, a study involving cesarean sections performed with 6.6 mg of hyperbaric bupivacaine showed that, in the sitting position, the anesthetic drug spread less in the cephalic direction (23). 

Patients in the lateral position used significantly fewer narcotics compared to those in the supine position, with no narcotics needed between 60 to 120 minutes post-operation, while the supine group required opioids within the first 60 minutes. This reduction helps mitigate side effects such as respiratory depression and nausea. Similarly, another study showed that cesarean sections in the lateral position resulted in faster onset of sensory and motor blocks, lower ephedrine use, and higher patient satisfaction compared to the sitting position (24). 

Multi-modal analgesia approaches have effectively minimized the reliance on opioid medications for postoperative pain management (19, 25). 

This study observed significant reductions in systolic blood pressure and heart rate in the lateral position compared to the supine position, with no significant differences in diastolic blood pressure or MAP. Hemodynamic stability was better in the lateral position, aligning with previous research showing less pronounced hemodynamic changes and lower blood pressures due to reduced cephalic spread of anesthetic (20). 

In a study with elderly patients, one group had spinal induction in the lateral position before switching to supine, while the other group was induced in the sitting position and then changed to supine after two minutes, both receiving 6.5 mg of hyperbaric bupivacaine. There were no significant differences in hemodynamic changes between the groups (26). 

In comparison, our study with higher doses of hyperbaric bupivacaine (15 - 20 mg), which showed notably lower blood pressure values in the lateral position, found that Fredman et al. observed no significant difference in MAP changes between the lateral and sitting positions with 10 mg of bupivacaine in older patients. This aligns with our MAP observations (4).

### 5.5. Study Limitations 

Limitations of our study include the subjective nature of pain assessments and the use of subjective tools to evaluate study endpoints. Future research should incorporate more objective measures to assess pain severity and relief, as well as explore additional factors influencing postoperative outcomes in patients undergoing spinal anesthesia with hyperbaric bupivacaine.

### 5.6. Conclusions 

Immediate transition from the sitting to lateral position following induction with hyperbaric bupivacaine enhances hemodynamic stability, optimizes drug distribution in the CSF, improves sensory block quality, prolongs postoperative analgesia, reduces opioid consumption, and enhances patient satisfaction. This approach holds promise for improving recovery outcomes and pain management in surgical settings.

### 5.7. The Message of the Study 

Pain management strategies should utilize 15 - 20 mg of hyperbaric 0.5% bupivacaine for spinal anesthesia induction and promptly transition PCNL candidates to the lateral position to enhance recovery outcomes and prolong analgesic duration.

## Data Availability

The dataset presented in the study is available upon request from the corresponding author during submission or after publication. The data are not publicly available because the processed information is fully reflected in the tables and figures, and does not require any raw data. Other data are in Persian and can be provided to any requester through correspondence.

## Clinical Trial Registration Code:

IRCT20221109056450N1.
